# The Clinical Impact of Change in the C-Reactive Protein/Albumin Ratio in Gastric Cancer Patients Who Receive Curative Treatment

**DOI:** 10.1007/s12029-023-00970-z

**Published:** 2023-10-02

**Authors:** Toru Aoyama, Yukio Maezawa, Itaru Hashimoto, Kentaro Hara, Keisuke Komori, Kazuki Otani, Keisuke Kazama, Sho Sawazaki, Masakatsu Numata, Shinnosuke Kawahara, Haruhiko Cho, Junya Morita, Kenki Segami, Mie Tanabe, Norio Yukawa, Aya Saito, Yasushi Rino, Takashi Ogata, Takashi Oshima

**Affiliations:** 1 Department of Surgery, Yokohama City University, Yokohama, Japan; 2 Department of Gastrointestinal Surgery, Kanagawa Cancer Center, Yokohama, Japan; 3 Department of Surgery, Tokyo Metropolitan Cancer and Infectious Diseases Center Komagome Hospital, Tokyo, Japan

**Keywords:** C-reactive protein/albumin ratio, Gastric cancer, Curative treatment

## Abstract

**Background:**

Recently, change in the C-reactive protein/albumin ratio (CAR) has become a promising prognostic marker in some malignancies. The aim of the present study was to evaluate the clinical impact of change in the CAR in gastric cancer patients who received curative resection.

**Method:**

The present study included 458 patients who underwent curative treatment for gastric cancer between 2013 and 2017. The prognosis and clinicopathological parameters were compared between patients who showed a high-change in CAR and those who showed a low-change in CAR.

**Results:**

The OS stratified by each clinical factor was compared using a log-rank test, and a significant difference was observed using a 0.05 change in CAR. When the patient background factors were compared between the high-change (change in CAR ≥ 0.05) and low-change (change in CAR < 0.05) groups, the median age, sex ratio, T factor, and N factor were similar. In the low-change group, the OS rates at 3 and 5 years after surgery were 94.1% and 87.6%, respectively, which amounted to a significant difference from the low-change group, with rates of 83.6 and 77.5% in the high-change group. In the low-change group, the RFS rates at 3 and 5 years after surgery were 90.1% and 85.1%, respectively, while those in the high-change group 77.6 and 75.2%. The univariate and multivariate analyses of factors associated with OS and RFS showed that the change in CAR was a significant prognostic factor.

**Conclusions:**

The change in CAR is a significant risk factor and promising prognostic factor for gastric cancer patients.

## Introduction

Gastric cancer is the fourth most common cancer and the second leading cause of cancer-related death in the world [1, 2]. The prognosis of gastric cancer is gradually improving with the improvement of minimally invasive surgery, perioperative care, and perioperative adjuvant treatment [3–5]. However, almost half of patients experience recurrence even after curative treatment [6, 7]. It is necessary to identify the prognostic factors and/or predictors of perioperative adjuvant treatment.

To date, several prognostic factors and predictors have been evaluated in gastric cancer. Recently, the nutrition status and systemic inflammation status have been shown to affect short- and long-term oncological outcomes [8]. Among them, the C-reactive protein/albumin ratio (CAR) is a promising prognostic factor. The CAR is identified as a non-specific marker of systemic inflammation. Some studies have shown that GC patients with a high CAR have a significantly poorer prognosis than those with a low CAR [9–11]. On the other hand, inflammation and nutritional status fluctuate dramatically. Thus, the CAR is variable at the diagnosis, as well as before and after gastrectomy. However, the optimal timing of the evaluation of the CAR is not well defined. In addition, one-point measurement of the CAR might not be reproducible. Considering these factors, we hypothesize that measurement of the change in CAR during treatment would have a greater clinical impact in gastric cancer treatment than the measurement of the CAR at a single point. To confirm our hypothesis, we evaluated the clinical impact of change in the CAR in gastric cancer patients who received curative treatment.

## Patients and Methods

### Patients

Patients were selected based on the medical records of consecutive patients who underwent curative resection for gastric cancer at Kanagawa Cancer Center from 2013 to 2017. The inclusion criteria were as follows: (i) histologically proven adenocarcinoma, (ii) clinical stage I–III disease as evaluated according to the 15th Edition of the General Rules for Gastric Cancer published by the Japanese Gastric Cancer Association [12], (iii) curative gastrectomy as a primary treatment for gastric cancer, (iv) complete (R0) resection of gastric cancer with radical lymph node dissection, and (v) ≥ 16 harvested lymph nodes. We excluded the patients who underwent R1 or R2 resection and those who did not measure CAR during perioperative periods.

### Surgical Procedure and Adjuvant Treatment

All patients underwent gastrectomy with nodal dissection. D1+ nodal dissection was performed for those with clinical stage IA disease, and D2 dissection was performed for those with clinical stage ≥ IB disease. Patients diagnosed with pathological II or III disease received S-1-based adjuvant chemotherapy within 6 weeks after surgery [13–15].

### Measurement of the CAR

The CAR was calculated as the serum CRP level (mg/dl) divided by the serum albumin level (g/dl) measured 1 week before surgery and 1 month after surgery. The change in CAR was defined as follows: Change in CAR = CAR at 1 month after surgery − CAR at 1 week before surgery.

### Follow-up

Patients were followed-up at outpatient clinics. Hematological tests and physical examinations were performed at least every 3 months for 5 years. The carcinoembryonic antigen and CA19 9 tumor marker levels were also checked at least every 3 months for 5 years. Patients underwent a computed tomography examination every 6–12 months until 5 years after surgery.

### Evaluations and Statistical Analyses

The significance of differences in the change in CAR and clinicopathological parameters was determined using the chi-squared test. The Kaplan–Meier method was used to calculate the overall survival and recurrence-free survival curves. Univariate and multivariate survival analyses were performed using a Cox proportional hazards model. We used stepwise methods for multivariate analysis in the present study. *P* values of < 0.05 were considered to indicate statistical significance. The SPSS software program (v27.0 J Win; IBM, Armonk, NY, USA) was used for all statistical analyses. This study was approved by the IRB of Kanagawa Cancer Center.

## Results

### Patients

We investigated 458 gastric cancer patients in the present study. Among them, 307 patients were male, and 322 patients were > 65 years of age. Sixty-seven patients had postoperative complications. The median CRP value before treatment was 0.07 (range: 0.01–1.54), the median albumin value was 4.1 (range: 2.3–5.1), and the median CAR was 0.01 (range: 0.002–1.543). The median CRP value after treatment was 0.1 (range: 0.01–16.43), the median albumin value was 4. (range: 1.4–4.9), and the median CAR value was 0.025 (range: 0.002–5.476).

### Survival Analysis

**Fig. 1:**
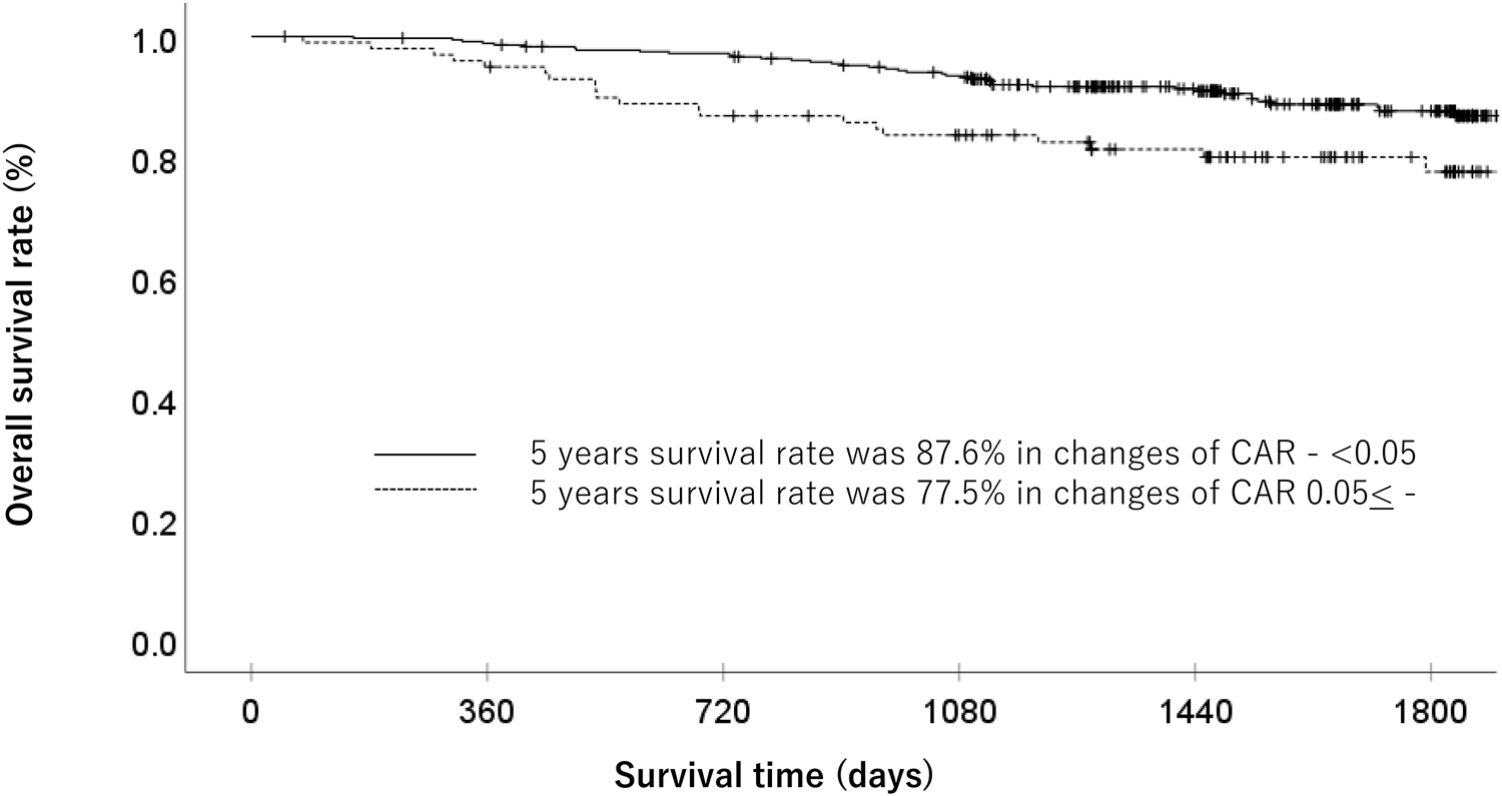
Overall survival in gastric cancer patients with high change in CAR (≥ 0.05) and low change in CAR (< 0.05)

**Fig. 2:**
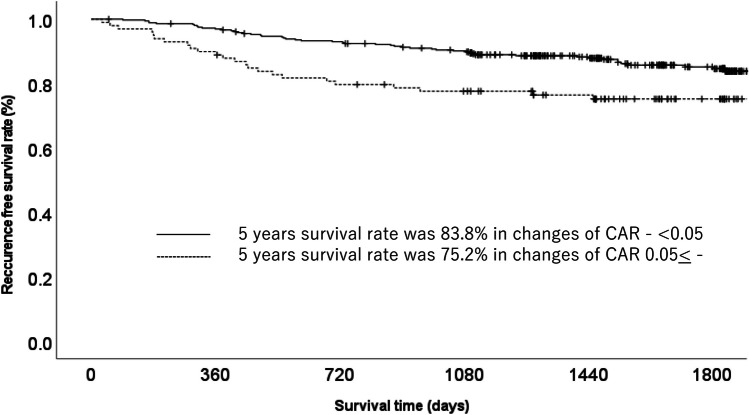
Recurrence-free survival in gastric cancer patients with high change in CAR (≥ 0.05) and low change in CAR (< 0.05)

The OS stratified by each clinical factor was compared using a log-rank test, and a significant difference was observed using a change in CAR of 0.05 (Table 1). When the patient background factors were compared between the high-change (change in CAR ≥ 0.05) and low-change (change in CAR < 0.05) groups, the median age, sex ratio, T factor, and N factor were similar. Each clinicopathological factor was categorized as shown in Table 2 and analyzed for its prognostic significance. The univariate and multivariate analyses for OS showed that pathological N factor, vascular invasion, and change in CAR were significant prognostic factors. The change in CAR was therefore selected for the final multivariate analysis model. In the low-change group, the OS rates at 3 and 5 years after surgery were 94.1% and 87.6%, respectively, while those in the high-change group were 83.6 and 77.5%, which amounted to a significant difference. The OS curves are shown in Fig. 1. The univariate and multivariate analyses of factors associated with RFS showed that the change in CAR was a significant prognostic factor. The change in CAR was selected as a significant prognostic factor for the final multivariate analysis model (Table 3). In the low-change group, the RFS rates at 3 and 5 years after surgery 90.1% and 85.1%, respectively, while those in the high-change group were 77.6 and 75.2%. The RFS curves are shown in Fig. 2.

**Table 1:** Patient characteristics

Characteristics	No. of patients (%)	1 year OS rate (%)	3-year OS rate (%)	5-year OS rate (%)	*p* value
Age (years)					0.047
< 65	136 (29.7%)	99.3	97.0	91.2	
≥ 65	322 (70.3%)	97.5	89.3	83.0	
Gender					0.059
Man	307 (67.0%)	98.0	90.4	83.2	
Woman	151 (37.0%)	98.0	93.9	90.0	
Pathological type					0.512
Intestinal	216 (47.2%)	98.1	93.0	86.4	
Diffuse	242 (52.8%)	97.9	90.2	84.7	
UICC T status					< 0.001
T1	285 (62.2%)	98.9	96.1	92.4	
T2 to T3	173 (37.8%)	96.5	84.7	73.8	
Lymph node metastasis					< 0.001
Negative	332 (72.5%)	98.5	96.0	91.9	
Positive	126 (27.5%)	96.8	79.7	67.8	
Lymphatic invasion					< 0.001
Negative	316 (69.0%)	98.1	94.9	91.3	
Positive	142 (31.0%)	97.9	84.9	72.1	
Vascular invasion					< 0.001
Negative	260 (56.8%)	99.2	97.7	93.7	
Positive	198 (43.2%)	96.4	84.1	74.4	
Change of CAR					0.008
< 0.05	359 (78.4%)	98.9	94.1	87.6	
0.05 ≤	99 (21.6%)	94.9	83.6	77.5	
Postoperative complications					0.653
Yes	67 (14.6%)	97.0	89.3	83.0	
No	391 (85.4%)	98.2	92.0	85.9	

*OS* Overall survival, *UICC* Union for International Cancer Control, *CAR* C-reactive protein albumin ratio

**Table 2:** Uni and multivariate Cox proportional hazard analysis of clinicopathological factors for overall survival

Factors	No	Univariate analysis			Multivariate analysis		
		*OR*	95% *CI*	*p* value	*OR*	95% *CI*	*p* value
Age (years)				0.051			
< 65	136	1.000					
65 ≦	322	1.920	0.997–3.698				
Gender				0.063			
Woman	151	1.000					
Man	307	1.794	0.969–3.321				
Pathological type							
Intestinal	216	1.000		0.512			
Diffuse	242	1.188	0.710–1.986				
UICC T status				< 0.001			
T1	285	1.000					
T2–T4	173	3.932	2.277–6.793				
Lymph node metastasis				< 0.001			< 0.001
Negative	332	1.000			1.000		
Positive	126	4.530	2.691–7.625		2.958	1.705–5.132	
Change of CAR				0.009			0.046
< 0.05	359	1.000			1.000		
0.05 ≤	99	2.054	1.198–3.523		1.733	1.009–2.978	
Lymphatic invasion				< 0.001			
Negative	316	1.000					
Positive	142	3.613	2.147–6.079				
Vascular invasion				< 0.001			< 0.001
Negative	260	1.000			1.000		
Positive	198	4.907	2.690–8.948		3.242	1.714–6.129	
Postoperative complications				0.653			
No	391	1.000					
Yes	67	1.169	0.592–2.308				

*UICC* Union for International Cancer Control

**Table 3:** Uni and multivariate Cox proportional hazards analysis of clinicopathological factors for recurrence free survival

Factors	No	Univariate analysis			Multivariate analysis		
		*OR*	95% *CI*	*p* value	*OR*	95% *CI*	*p* value
Age (years)				0.059			
< 65	136	1.000					
65≦	322	1.725	0.979–3.041				
Gender				0.347			
Woman	151	1.000					
Man	307	1.275	0.769–2.113				
Pathological type				0.377			
Intestinal	216	1.000					
Diffuse	242	1.230	0.777–1.949				
UICC T status				< 0.001			
T1–T2	285	1.000					
T3–T4	173	3.326	2.071–5.341				
Lymph node metastasis				< 0.001			< 0.001
Negative	332	1.000			1.000		
Positive	126	4.523	2.847–7.185		2.555	1.491–4.378	
Change of CAR				0.008			0.020
< 0.05	359	1.000			1.000		
0.05 ≤	99	1.942	1.193–3.159		1.792	1.098–2.927	
Lymphatic invasion				< 0.001			0.021
Negative	316	1.000			1.000		
Positive	142	3.807	2.392–6.060		1.903	1.101–3.291	
Vascular invasion				< 0.001			0.023
Negative	260	1.000			1.000		
Positive	198	3.615	2.194–5.956		1.909	1.092–3.336	
Postoperative complications				0.264			
Yes	391	1.000					
No	67	1.394	0.779–2.494

### Postoperative Course of the Low-Change and High-Change Groups

When the postoperative course of the two groups was compared, there were some differences in the incidence of postoperative surgical complications and the incidence of other-cause deaths. The incidence of postoperative complications was 32.3% (32/99) in the high-change group and 9.7% (35/359) in the low-change group. The incidence of surgical complications in the high-change group was significantly higher than that in the low-change group (*p* = 0.009). The incidence of other causes of death was in the high-change and low-change groups was 10.1% (10/99) and 3.6% (13/359), respectively, which amounted to a significant difference (*p* < 0.001). Furthermore, there was a significant difference between the two groups in the site of first relapse at lymph node metastasis (Table 4).

**Table 4:** Patterns of recurrence between the patients with C-reactive protein albumin ratio < 0.05 and those with C-reactive protein albumin ratio 0.05 ≤

Recurrence site	All cases (*n* = 458)		C-reactive protein albumin ratio				*p* value
			< 0.05 (*n* = 359)		0.05 ≤ (*n* = 99)		
	Number	%	Number	%	Number	%	
Peritoneal	17	3.7	14	3.9	3	3.0	0.685
Hematological	16	3.5	11	3.1	5	5.1	0.341
Lymph node	13	2.8	5	1.4	8	8.1	< 0.001
Local site	4	0.8	4	1.1	0	0	0.291

## Discussion

The aim of the present study was to clarify the clinical impact of change in CAR in gastric cancer patients who received curative treatment. The major finding is that a change in CAR is a significant risk factor in gastric cancer patients. In addition, a higher change in CAR is related to postoperative complications and lymph node recurrence. Therefore, a change in CAR is a promising prognostic factor for gastric cancer patients.

In the present study, the high-change CAR (≥ 0.05) group had a significantly poorer prognosis in comparison to the low-change CAR (< 0.05) group (hazard ratio: 1.733, 95% confidence interval: 1.009–2.978, *p* = 0.0049). Moreover, the 5-year OS rates of the high-change CAR group and low-change CAR group were 77.5% and 87.6%, respectively. Although limited studies have evaluated the clinical impact in malignancies, similar results were observed in previous studies. Oshima et al. evaluated the changes and prognostic impact of inflammatory nutritional factors during neoadjuvant chemoradiation therapy in 49 patients with resectable pancreatic cancer [16]. They found that high change in CAR (≥ 0.077) after chemoradiation therapy were significantly associated with shorter OS. The median OS was 23.1 months in patients with a high change in CAR and 64.1 months in those with a low change in CAR. In addition, change in CAR after chemoradiation therapy were a prognostic factor (hazard ratio: 5.1842, *p* = 0.0036). Moreover, Ikoma et al. evaluated the clinical impact of change in CAR in 97 gastric cancer patients who received immune checkpoint inhibitor therapy [17]. They found that patients with low change in CAR (≤ 0.01) during immune checkpoint inhibitor therapy had better survival than those with high change in CAR (> 0.01). The median OS was 9.4 months in the patients with low change in CAR and 4.5 months in those with high change in CAR; the difference was statistically significant (*p* = 0.002). In addition, the univariate and multivariate analyses showed that low change in CAR was a prognostic factor (*HR* 0.59; 95% *CI* 0.37–0.93; *p* = 0.002). Considering our results and previous reports, the change in CAR during cancer treatment might be a promising prognostic factor.

Recently, the change in CAR during treatment might be not only a promising prognostic factor but also a promising nutritional assessment tool for nutritional intervention. To date, there have been various nutritional/anti-inflammatory treatment studies for malignancy patients during cancer treatment [18, 19]. However, limited studies have shown only positive results, and there are no definitive nutritional/anti-inflammatory treatments. One possible reason is the difficulty of setting an endpoint. Although previous studies, including our studies, focused on body weight or lean body mass, the effect of nutritional/anti-inflammation treatment during cancer treatment on weight and muscle was much smaller than expected, and the difference may not have been detectable. On the other hand, the change in CAR might indicate small change in nutritional/anti-inflammatory treatment during cancer treatment. For example, Silva et al. conducted a clinical trial to evaluate the systemic inflammatory response to eicosapentaenoic acid (EPA) in 29 colorectal cancer patients [20]. The patients who were assigned to the supplemental group received 2 g of fish oil containing 600 mg of EPA and docosahexaenoic acid (DHA). When the change in CAR between baseline and 9 weeks after chemotherapy was compared, the patients who received EPA showed a clinically relevant decrease in their C-reactive protein/albumin ratio (*p* = 0.005).

They found that low doses of fish oil supplementation can positively modulate the nutritional status and C-reactive protein/albumin ratio. Considering these factors, change in the CAR might become promising predictive markers of nutritional/anti-inflammatory treatment during cancer treatment.

To introduce change in CAR as a parameter in daily clinical practice, it is necessary to set the optimal cutoff value. In the present study, we set the cutoff value of change in CAR at 0.05 according to the 1-, 3-, and 5-year survival rates. Several values of the change in CAR have been described in previous studies. Oshima et al. set the cutoff value of change in CAR at 0.077, and Ikoma et al. set it at 0.01. These differences might be due to the following reasons. First, the number of patients and patient background factors were different. Our study (*n* = 458) evaluated resectable gastric cancer, Ikoma et al. evaluated unresectable gastric cancer (*n* = 97), and Oshima et al. evaluated pancreatic cancer (*n* = 49). Second, the methods used to evaluate change in CAR were different. Our study evaluated the cutoff value of change in CAR according to the survival rate, while other studies evaluated the cutoff value of change in CAR according to a receiver operating characteristic curve. These differences might have affected the cutoff value. Further studies are needed to establish the optimal evaluation method and optimal cutoff value of change in CAR.

The present study was associated with some limitations. First, this was a retrospective study with a small sample size from a single institution. Therefore, our study might have a selection bias. Second, there might be time bias in the present study. Our study included data from 2012 to 2017. During this period, perioperative care and perioperative adjuvant treatment were improved. Third, in the present study, the incidence of other-cause death was significantly higher in the high-change group than in the low-change group. However, the reason for this issue is unclear. Considering these factors, our results need to be validated in another large cohort.

In conclusion, change in CAR during treatment is a significant risk factor and in gastric cancer patients and is therefore a promising prognostic factor. In addition, the changes of inflammation status during perioperative periods might be a promising prognostic factor for gastrointestinal cancer patients.

## Data Availability

The anonymized data used and/or analyzed during the current study are available from the corresponding author upon reasonable request.
